# Role of *CpALS4790* and *CpALS0660* in *Candida parapsilosis* Virulence: Evidence from a Murine Model of Vaginal Candidiasis

**DOI:** 10.3390/jof6020086

**Published:** 2020-06-12

**Authors:** Marina Zoppo, Fabrizio Fiorentini, Cosmeri Rizzato, Mariagrazia Di Luca, Antonella Lupetti, Daria Bottai, Marisa Colone, Annarita Stringaro, Flavia De Bernardis, Arianna Tavanti

**Affiliations:** 1 Department of Biology, University of Pisa, 56127 Pisa, Italy; Fabrizio.Fiorentini@iit.it (F.F.); mariagrazia.diluca@unipi.it (M.D.L.); daria.bottai@unipi.it (D.B.); 2 Department of Translational Research and New Technologies in Medicine and Surgery, University of Pisa, 56127 Pisa, Italy; cosmeri.rizzato@unip.it (C.R.); antonella.lupetti@med.unipi.it (A.L.); 3 National Center for Drug Research and Evaluation, Istituto Superiore di Sanità, 00161 Rome, Italy; marisa.colone@iss.it (M.C.); annarita.stringaro@iss.it (A.S.); 4 Department of Infectious Diseases, Istituto Superiore di Sanità, 00161 Rome, Italy; flavia.debernardis@iss.it

**Keywords:** *C. parapsilosis*, *ALS*, SAT1-flipper cassette, adhesion, HBECs, murine vaginal candidiasis

## Abstract

The *Candida parapsilosis* genome encodes for five agglutinin-like sequence (Als) cell-wall glycoproteins involved in adhesion to biotic and abiotic surfaces. The work presented here is aimed at analyzing the role of the two still uncharacterized *ALS* genes in *C. parapsilosis, CpALS4790* and *CpALS0660*, by the generation and characterization of *CpALS4790* and *CpALS066* single mutant strains. Phenotypic characterization showed that both mutant strains behaved as the parental wild type strain regarding growth rate in liquid/solid media supplemented with cell-wall perturbing agents, and in the ability to produce pseudohyphae. Interestingly, the ability of the *CpALS0660* null mutant to adhere to human buccal epithelial cells (HBECs) was not altered when compared with the wild-type strain, whereas deletion of *CpALS4790* led to a significant loss of the adhesion capability. RT-qPCR analysis performed on the mutant strains in co-incubation with HBECs did not highlight significant changes in the expression levels of others *ALS* genes. In vivo experiments in a murine model of vaginal candidiasis indicated a significant reduction in CFUs recovered from BALB/C mice infected with each mutant strain in comparison to those infected with the wild type strain, confirming the involvement of CpAls4790 and CpAls5600 proteins in *C. parapsilosis* vaginal candidiasis in mice.

## 1. Introduction

The agglutinin-like sequence (Als) family encodes for cell-wall glycoproteins involved in fungal adhesion to both biotic and abiotic surfaces [1]. Since the description of the first *ALS* gene more than two decades ago [2], the *ALS* gene family has been extensively studied in *Candida albicans*, where eight Als members have been identified and characterized [1]. The *Candida parapsilosis* species complex encompasses three closely related species named *Candida parapsilosis*, *Candida orthopsilosis* and *Candida metapsilosis* [3]. Among them, *C. parapsilosis* has recently gained importance as one of the leading causes of invasive candidiasis among non-*albicans* species [4,5]. Moreover, metagenomic studies proved the presence of *C. orthopsilosis* and *C. metapsilosis* in different body sites of newborns [6] and in the oral cavity of human subjects, respectively [7]. While genome sequencing allowed the identification of the *ALS* gene family in the *C. parapsilosis* species complex [8,9,10], its combination with long-read sequencing technologies has led to the generation of a more accurate genome assembly, allowing the sequencing of misassembled repetitive regions characteristic of the *ALS* genes [11,12]. The *C. parapsilosis* genome harbors five *ALS* genes: four located on chromosome 4 and called *CpALS4770*, *CpALS4780*, *CpALS4790* and *CpALS4800*, respectively, and the remaining one, named *CpALS0660*, located on chromosome 5. Despite sharing essential traits with the well-characterized *C. albicans* orthologues, structure analysis of the five *C. parapsilosis* Als proteins, as well as of the other members of the *C. parapsilosis* species complex, revealed specific diverging features. While CpAls4780 and CpAls4790 are the only *C. parapsilosis* proteins retaining the NT/T/TR/CT organization typical of *C. albicans* Als proteins [1], the remaining proteins have short imperfect repeated sequences, such as a SSSEPP motif and/or a GSGN+ motif, instead of the tandem repeat region. As part of a larger effort aimed at dissecting the role of each *C. parapsilosis ALS* gene, the contribution of *CpALS4800, CpALS4770* and *CpALS4780* have been demonstrated through the generation of mutant strains using either the SAT1-flippler cassette system or the CRISPR-Cas9 gene editing technology [13,14]. While adhesion experiments preformed on human buccal epithelial cells (HBECs) indicated a direct involvement of *CpALS4800* and *CpALS4770*, all three *ALS* genes characterized so far demonstrated a role in *C. parapsilosis* pathogenesis when tested in a murine model of urinary tract or vaginal infection [13,14]. Moreover, gene inactivation of *ALS* genes performed in a *C. parapsilosis* clinical isolate highlighted an important role of *CpALS4770* to biofilm formation on abiotic surfaces [13].

In the present study, the contribution of the two remaining uncharacterized *ALS* genes, *CpALS4790* and *CpALS0660*, was investigated through the generation and the in vitro/in vivo characterization of homozygous *CpALS4790* and *CpALS0660* single mutants. Both mutants were obtained in a two-step approach using the SAT1-flipper cassette strategy. *CpALS4790* and *CpALS0660* null mutant strains underwent a set of phenotypical tests aimed at evaluating growth ability, cell-wall integrity, morphogenesis, adhesion to HBECs and gene expression level fluctuations within the *ALS* family. Finally, *CpALS4790* and *CpALS0660* mutants were tested in a murine model of vaginal candidiasis to assess the role of CpAls4790 and CpAls0660 proteins in host–*C. parapsilosis* interactions.

## 2. Materials and Methods

### 2.1. Strains and Plasmids Used in This Study

*C. parapsilosis* parental and mutant strains generated in this study are listed in Table S1. The strains were grown in YPD medium (10 g yeast extract, 20 g peptone, 20 g dextrose, 15 g agar per liter (CondaLab, Madrid, Spain). Following SAT1-flipper cassette transformation, *C. parapsilosis* transformants were grown on YPD agar plates supplemented with 100 μg/mL of Nourseothricin (NTC, Werner BioAgents, Jena, Germany) (YPD-NTC). Recycling of the SAT1-flipper cassette was performed in Yeast Nitrogen Base broth (YNB, Fisher Scientific Italia, Rodano, Milan, Italy) supplemented with maltose (20 g per liter). Competent *Escherichia coli* 5-alpha F’I^q^ cells (New England Biolabs, Ipswich, MA, USA) were used for bacterial cloning experiments. *E. coli* cells were routinely grown in Luria Bertani (LB) liquid medium (Fisher Scientific, Waltham, Massachusetts, US). When required, 100 μg/mL Ampicillin (Amp, Sigma Aldrich, Milan, Italy) was added to the medium (LB-Amp).

### 2.2. Construction of CpALS4790 and CpALS0660 Disruption Cassettes

The pSFS2 plasmid harbouring the SAT1-flipper cassette [15] was used to generate the *CpALS4790* and *CpALS0660* disruption cassettes. Upstream and downstream homology regions of *CpALS4790* and *CpALS0660* were amplified from the genomic DNA of the reference strain of *C. parapsilosis* ATCC 22019 using primers containing ApaI/XhoI and SacII/SacI restriction sites, respectively (HOM6UPF/HOM6UPR and HOM6DWF/HOM6DWR for *CpALS4790* and HOM10UPF/HOM610PR and HOM10DWF/HOM10DWR for *CpALS0660*, Table S2). The amplified 3′ HOM regions (3′HOMCpALS4790, 570 bp from +6864 to +7434 for *CpALS4790* and 3′HOMCpALS0660 596 bp from +2891 to +3487 for *CpALS0660*) were purified using the QIAquick PCR Purification Kit (Qiagen, Hilden, Germany). Plasmid pSFS2 and downstream homology regions were digested with SacII and SacI (New England Biolabs, Ipswich, MA, USA) and ligated yielding plasmids p3HOMCpALS4790 and p3HOMCpALS0660, respectively. Competent *E. coli* 5-alpha F’Iq cells were used for bacterial transformation by heat shock and plated on LB-Amp solid media. The amplified 5′ HOM regions (5′HOMCpALS4790, 647 bp from −250 to +397 for *CpALS4790* and 5′HOMCpALS0660a, 630 bp from −743 to −113 for *CpALS0660*) were purified as described above and ligated respectively with plasmids p3HOMCpALS4790 and p3HOMCpALS0660 previously double digested with ApaI and XhoI (New England Biolabs, Ipswich, MA, USA). The ligation mixture was transformed in competent *E. coli* cells and recombinant colonies were selected on LB-Amp solid media as previously described. The resulting plasmids harbouring the *CpALS4790* and *CpALS0660* disruption cassettes were named p35HOMCpALS4770 and p35HOMCpALS0660a, respectively.

Following the inactivation of the first *CpALS0660* allele, an alternative version of the disruption cassette was created for the deletion of the remaining allele. Briefly, primers HOM10UPF1 and HOM10UPR1, harbouring the ApaI and XhoI sites respectively, were used for the amplification of a more internal upstream homology region of *CpALS0660* (5′HOMCpALS0660b, 503 bp, from −111 to +392). The amplified fragment was purified as previously described and digested, together with p3HOMCpALS0660, with ApaI and XhoI. Ligation of these two components led to the creation of p35HOMCpALS0660b. 

**Figure 1:**
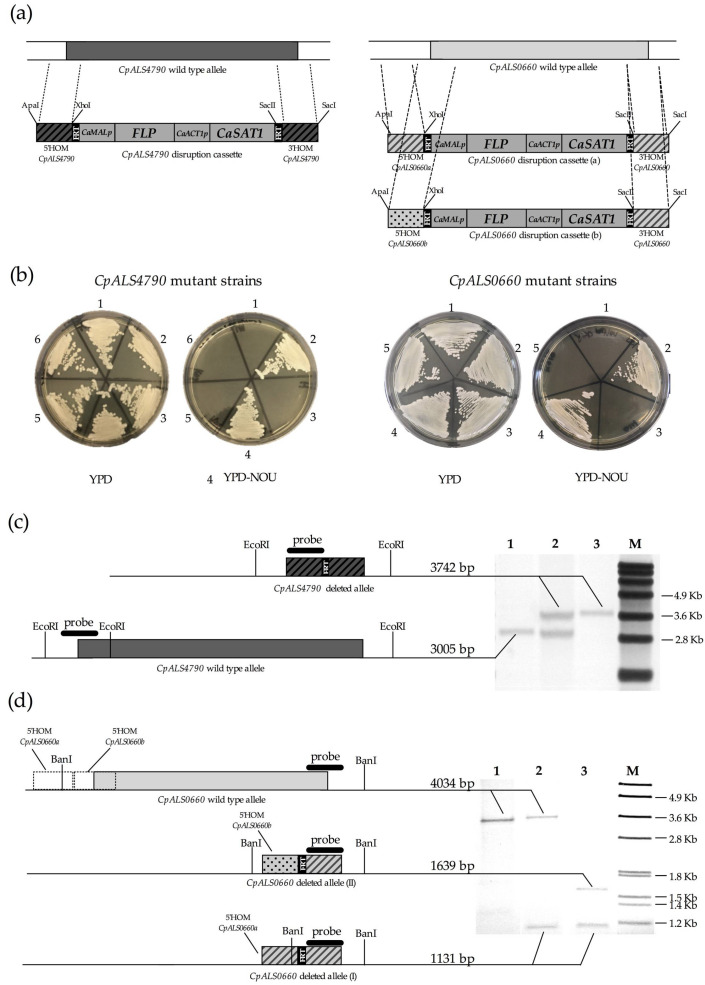
Gene disruption strategy and southern blot analysis of the mutant panel. (**a**) Left panel: *CpALS4790* disruption cassette harbouring the two homology regions (5′HOMCpALS4790 and 3′HOMCpALS4790) required for the correct integration in the target locus. Right panel: *CpALS0660* disruption cassette harbouring the homology regions (5′HOMCpALS0660, 5′HOMCpALS0660b and 3′HOMCpALS0660) required for the correct integration in the target locus. The following abbreviations are used in the description of disruption cassettes: *CaMAL2p*: *C. albicans* maltose-inducible promoter; *CaFLP*: *C. albicans*-adapted *FLP* gene encoding the site-specific recombinase *FLP*; *CaACT1p*: *C. albicans* actin promoter; *CaSAT1*: *C. albicans*-adapted Nourseothricin resistance marker; FRT: (FLP-recognition target) minimal recombination target sites of the *FLP* recombinase. (**b**) Left panel: *CpALS4790* mutant collection plated on YPD and YPD-Nou agar plates and grown for 48 h at 30°C. 1: ATCC 22019; 2: CpALS4790HC; 3: CpALS4790H; 4: CpALS4790KOC; 5: CpALS4790KO; 6: ATCC 22019. Right panel: *CpALS0660* mutant collection plated on YPD and YPD-Nou agar plates and grown for 48 h at 30 °C. 1: ATCC 22019; 2: CpALS0660HC; 3: CpALS0660H; 4: CpALS0660KOC; 5: CpALS0660KO. (**c**) Schematic representation of the Southern blot strategy used to check the genotype of the *CpALS4790* mutant collection. 1: ATCC 22019; 2: CpALS7490H; 3: CpALS4790KO; M: Roche Dig Labelled Marker VII. (**d**) Schematic representation of the Southern blot strategy used to check the genotype of the *CpALS0660* mutant collection. 1: ATCC 22019; 2: CpALS0660H; 3: CpALS0660KO; M: Roche Dig Labelled Marker VII.

Plasmids p35HOMCpALS4790, p35HOMCpALS0660a and p35HOMCpALS0660b were double digested with ApaI and SacI yielding the following disruption cassettes: one for the *CpALS4790* gene deletion (*CpALS4790* disruption cassette) and two for the *CpALS0660* gene deletions (*CpALS0660* disruption cassettes (a) and (b)) (Figure 1a). 

### 2.3. C. parapsilosis Transformation and Screening of the Mutant Collection

*C. parapsilosis* reference strain ATCC 22019 was transformed through electroporation as previously described [13,14,16,17]. After each round of transformation the correct integration of the disruption cassette in the *CpALS4790* locus was checked by PCR using primers ZOP2F-CpALS6ER (Table S2) leading to the identification of the heterozygous mutant strain named CpALS4790HC (Table S1). Similarly, the *CpALS0660* heterozygous mutant strain, named CpALS0660HC (Table S1), was selected through PCR by checking the correct integration of the disruption cassette in the *CpALS0660* locus with primers CpALS10EF-But237 (Table S2). The SAT1-flipper cassette was excised from the genome of the heterozygous mutant strains as previously described [16], leading to the generation of the CpALS4790H and CpALS0660H strains (Table S2). Briefly, to allow the recycling of the SAT1-flipper cassette, *C. parapsilosis* mutant strains were inoculated in 10 mL of Yeast Nitrogen Base minimal medium (Fisher Scientific Italia, Rodano, Milan, Italy) supplemented with 20 g/L of maltose (YNB-MAL) for 24–48 h. Activation of the *CaMAL2p* promoter and the consequent expression of the *FLP* recombinase resulted in the recognition of the FRT sites and in the excision of the SAT1-flipper cassette. Due to the orientation of the FRT sites, only one FRT was left between the upstream and the downstream homology regions. Following incubation in YNB-MAL medium, the cell suspension was diluted and seeded on YPD plates. Colonies were then replica-plated on YPD-NTC agar in order to identify the clones that lost the SAT1-flipper cassette and, therefore, the ability to grow in presence of Nourseothricin. The deletion of the remaining allele of *CpALS4790* and *CpALS0660* was obtained with a second round of transformation and *C. parapsilosis* recombinant colonies were screened as described above. The selected null mutant clones, named CpALS4790KOC and CpALS0660KOC (Table S1), respectively, underwent cassette recycling as described above yielding CpALS4790KO and CpALS0660KO null mutant strains (Table S1).

### 2.4. Southern Blot Analysis

In order to check the correct construction of the *CpALS4790* and *CpALS0660* mutant collection, total DNA was isolated and digested with EcoRI and BanI (New England Biolabs, Ipswich, MA, USA), respectively. Southern blot analysis was performed according to the guidelines of the DIG Application Manual for Filter Hybridization (www.roche-applied-science.com, Roche Diagnostic, Milan, Italy). The 5′ homology region of *CpALS4790* (647 bp) and the 3′ homology region of *CpALS0660* (596 bp) were used as hybridization probes and incubated at 44 °C and 45 °C, respectively. Following hybridization, an anti-digoxigenin antibody, able to recognize the labelled probe, and conjugated with the alkaline phosphatase enzyme, was added to the nylon membrane. Signal detection was performed through the addition of the chromogenic substrate NBT/BCIP (Roche S.p.A, Monza, Italy), which yielded an intense, insoluble black-purple precipitate when reacted with alkaline phosphatase.

### 2.5. Growth Ability and Morphogenesis

The growth ability of the *CpALS4790* and *CpALS0660* mutant collection was analysed in YPD medium. Briefly, *Candida* cells were inoculated in 10 mL of YPD broth and incubated overnight at 37 °C with shaking. Following incubation, cells were diluted 1:100 in 100 mL of YPD broth and incubated at 37 °C with shaking. The optical density (OD_600_) was monitored every 2 h for 24 h. A set of conventional phenotypic tests were run on the mutant strains generated. The susceptibility of the panel of wild type and mutant strains to cell-wall and membrane perturbing agents was investigated as previously described [13,14,17]. Briefly, *C. parapsilosis* strains were grown overnight in YPD liquid medium and serial dilution were set up from 10^7^ cells/mL to 10 cells/mL in sterile water. An aliquot of 10 μL for each of the dilutions prepared was spotted on YPD agar plates supplemented with the following compounds: 1 mg/L congo red (CR, Sigma Aldrich, Milan, Italy), 20 mg/L calcofluor white (CFW, Sigma Aldrich, Milan, Italy) and 5 mM caffeine (CAFF, Merck, Darmstadt, Germany). All compounds were tested at sub-fungicidal concentrations. Plates were incubated at 30 °C and 37° C for 48 h. The ability to undergo morphogenesis was assessed, incubating a suspension of 10^6^ cell/mL for each strain in YPD medium supplemented with 10% fetal bovine serum (FBS) at 37 °C for 24 h in polystyrene 24-well microtiter plates (BD Biosciences, San Jose, CA, USA) [13,14,17]. Following incubation, 10 μL of the suspension was analysed at 400× magnification in order to evaluate the presence of pseudohyphae. *C. albicans* SC5314 strain was used as positive control for hyphal production.

### 2.6. Adhesion on Human Buccal Epithelial Cells (HBECs)

The adhesion ability of the *CpALS4790* and *CpALS0660* mutant collection to human surfaces was evaluated in a model of co-incubation with human buccal epithelial cells (HBECs) according to a previously established method [13,14,16,17,18]. The donor of exfoliated buccal cells signed an informed consent in accordance with the Declaration of Helsinki. Local ethical Committee (Committee on Bioethics of the University of Pisa) approval was received for this set of experiments (Ref. no. 6/2019, Prot. No.0012910/2019). The number of yeast adherent to 100 HBECs was counted for each *C. parapsilosis* analyzed strain. The mean adhesion index obtained for 4 independent experiments, each performed in triplicate, was then calculated as previously described [14]. Statistical analysis was performed using One-way ANOVA followed by Bonferroni’s post-hoc test.

### 2.7. Quantitative Analysis of CpALS Genes Expression in Co-Incubation with HBECs

The expression level of *C. parapsilosis ALS* genes following co-incubation with HBECs was performed by real-time reverse transcription PCR (Real time RT-PCR) as previously described [13,14,16]. Following a 45-min incubation, the suspension of yeast and buccal cells was recovered and total RNA was extracted using the Nucleospin RNA kit (Macherey Nagel, Düren, Germany), according to the manufacturer’s instructions. Single-stranded complementary DNA synthesis was performed with random primers on 1 μg of total RNA using the Reverse Transcription System kit (Promega, Milano, Italy), according to the manufacturer’s instructions. qRT-PCR was performed using the primers listed in Table S2. Actin was used as a housekeeping gene for reference. Real time PCR mixture (20 μL) contained 1 μL of cDNA, 10 μL of SsoAdvancedTM universal SYBR^®^ Green supermix, 1 μL each of primers (final concentration 0.2 μM; Table S1) and 7 μL of sterile MilliQ water. Real time PCR was performed in 96 well plates on CFX96 Touch Real-Time PCR Detection System (BioRad, Segrate, Italy) (95 °C incubation for 60 s, followed by 40 cycles of 95 °C incubation for 5 s and 58 °C for 15 s). Actin was used as a housekeeping gene for reference. Transcription level of *ALS* genes was calculated using the formula of 2^-ΔΔCt^. RT-PCR results were evaluated by Repeated Measures ANOVA test, followed by Dunnett’s Multiple Comparison Test. A *p* value <0.05 was set as statistically significant.

### 2.8. Murine Model of Vaginal Candidiasis

The pathogenicity of *C. parapasilosis CpALS4790* and *CpALS0660* mutant collection was tested in a murine model of vaginal candidiasis. In vivo experiments were performed following the ethical protocol approved by the Committees on the Ethics of Animal Experiments of the “Istituto Superiore di Sanità”, Rome, Italy (Permit number: DM 227/2016-B dated 22/12/2016). For the experimental vaginal infection 5 female BALB/C mice (18–21 g; Harlan Laboratories, Lesmo, Italy) were injected subcutaneously with 0.02 mg of estradiol benzoate (Estradiolo, Amsa Farmaceutici srl, Rome, Italy) in 100 µL of saline, 48 h before inoculation with *Candida* strains and weekly thereafter. The animals were intravaginally infected with 10^6^ yeast cells in 20 µL of saline solution of each *C. parapsilosis* strain tested. The inoculum was dispensed into the vaginal cavity through a syringe equipped with a multipurpose calibrated tip (Combitip; PBI, Milan, Italy). The time course of infection was monitored for each animal by culturing 100 µl of undiluted and serially diluted vaginal fluids, taken at designated times (0, 1, 2, 5, 7, 14 and 21 days), and plated onto Sabouraud agar plates supplemented with chloramphenicol (50 mg/mL). CFU were enumerated after incubation at 30 °C for 48–72 h. Statistical analysis was performed using One-way ANOVA followed by Bonferroni’s post-hoc test.

### 2.9. Scanning Electron Microscopy (SEM)

Five days after vaginal infection, samples were taken and processed for scanning electron microscopy. This time point was selected based on previous experiments carried out in mice infected with other *C. parapsilosis* mutant strains, in which vaginal infection was clearly established by day 5 and mice infected with wild-type or mutant strains carried significantly different fungal burdens [13]. The samples were fixed with 2.5% (*v/v*) glutaraldehyde in 0.2 M cacodylate buffer. Cells were postfixed overnight with 1% (*w/v*) osmium tetroxide in 0.2 M cacodylate buffer at 4 °C. Samples were deposited on glass coverslips of 12 mm diameter for 30 min and were then dehydrated on an ethanol gradient and critical point dried in CO_2_. The coverslips were attached to aluminium stubs and gold coated by sputtering (SCD 040 Balzers device, Bal-Tec, Pfäffikon, Switzerland). The samples were examined with a scanning electron microscope FEI Quanta Inspect FEG, (Thermo Fisher Scientific, Waltham, MA, USA). 

## 3. Results

### 3.1. Construction and Genotypic Characterization of CpALS4790 and CpALS0660 Mutant Strains

To evaluate the role of *CpALS4790* and *CpALS0660* in *C. parapsilosis* virulence and pathogenicity, both alleles were deleted in *C. parapsilosis* ATCC 22019 reference strain, using the SAT1-flipper cassette strategy (Figure 1a). Two separate rounds of transformation, followed by SAT1-flipper cassette recycle, were performed in order to obtain heterozygous and null mutant strains of *CpALS4790* and *CpALS0660*. The correct integration of the disruption cassette in the *CpALS4790* or the *CpALS0660* locus was checked by PCR using primers Zop2F/CpALS6ER and CpALS10EF/But237, respectively (Figure S1a,b), leading to the identification of the CpALS4790HC and CpALS0660HC heterozygous strains in the first selection step, and the CpALS4790KOC and CpALS0660KOC null mutants in the second step. PCR analyses with primers CpALS6EF/CpALS6ER were performed to verify the correct excision of the SAT1-flipper cassette in the CpALS4790KO null mutant strain. The amplification of a 1.4 Kb fragment in the null mutant strain attested the effective deletion of the *CpALS4790* allele and the recycling of the SAT1-flipper cassette (Figure S1c). Similarly, the correct excision of the SAT1-flipper cassette was checked in the CpALS0660KO strain using primers CpALS10EF/CpALS10ER, yielding two fragments with different molecular weights (1.6 Kb and 2.1 Kb) due to their transformation with two different disruption cassettes (as described in paragraph 2.2). As expected, the same set of primers produced a 4.6 Kb fragment in the wild type strain (Supplementary Figure S1d)*. C. parapsilosis* mutant strains containing the SAT1-flipper cassette integrated in the genome (CpALS4790HC, CpALS4790KOC, CpALS0660HC and CpALS0660KOC) were Nourseothricin resistant and were maintained on YPD-NTC agar plates. All the other mutant strains in which the SAT1-flipper cassette was excised from the genome (CpALS4790H, CpALS4790KO, CpALS0660H and CpALS0660KO) were sensitive to Nourseothricin and were maintained on YPD agar plates (Figure 1b). Both *CpALS4790* and *CpALS0660* mutant sets were checked by Southern blot hybridization. Figure 1c,d illustrate a scheme of the *CpALS4790* and *CpALS0660* loci, respectively, in the different mutant strains obtained in this study and show the expected hybridization pattern for each isolate, confirming the correct genotype of both *CpALS4790* and *CpALS0660* mutant strains.

### 3.2. CpALS4790 and CpALS0660 Mutants Display Similar Growth Rates and Morphogenetic Properties as Compared to the Wild Type Strain 

**Figure 2:**
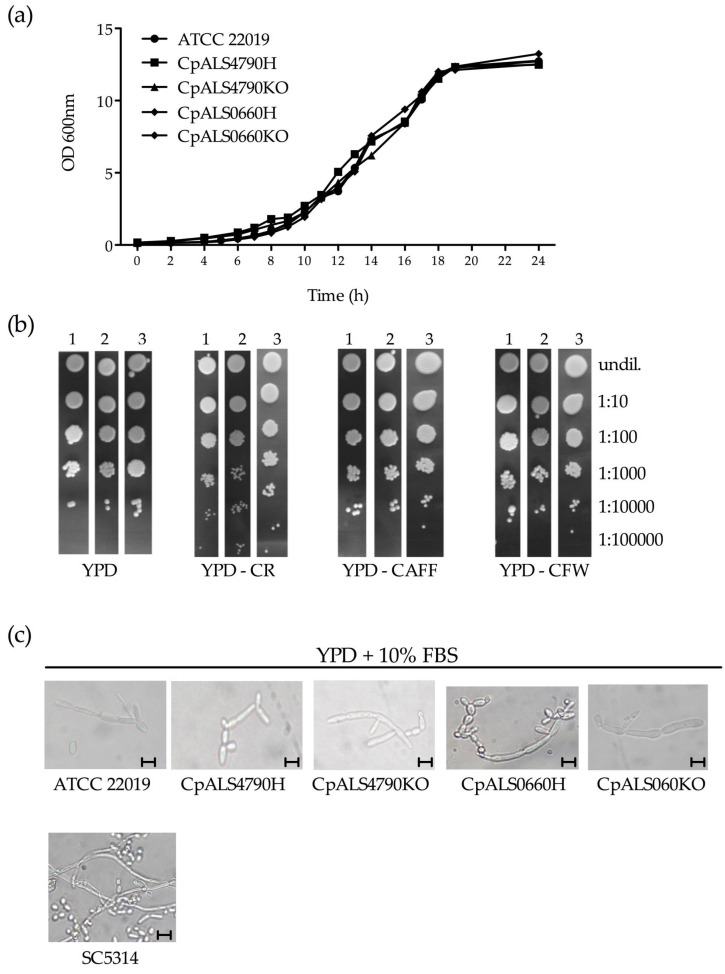
Growth ability and pseudohyphal formation. (**a**) Growth curve of the wild type and mutant collection of *CpALS4790* and *CpALS0660* in YPD medium at 37 °C. (**b**) Susceptibility to cell-wall perturbing agents of the *C. parapsilosis* strains was evaluated by spot assay, on YPD agar plates supplemented with the following compounds: congo red (CR, 1 mg/L), caffeine (CAFF, 5 mM), calcoflour white (CFW, 20 mg/L). Plates were incubated at 30°C for 48 h, and visually inspected. 1: wild type; 2: CpALS4790KO; 3: CpALS0660KO. Dilution factors are indicated on the side of the pictures. (**c**) Production of pseudohyphae by *CpALS4790* and *CpALS0660* mutant strains. *C. albicans* SC5314 was included as a positive control. Pseudohyphal production was induced in YPD broth supplemented with 10% FBS. The plates were incubated at 37 °C for 24 h. Following incubation, an aliquot of 10 μL from each culture was directly observed using an optical microscope at 400× magnification. Scale bars denote 10 μm.

The *CpALS4790* and *CpALS0660* mutant strains were phenotypically characterized for their ability to grow in YPD liquid medium at 37 °C. As shown in Figure 2a, no significant differences in the growth rates were observed between the wild type, heterozygous and null mutant strains. Similar results were obtained when growth was assessed on YPD agar plates supplemented with cell-wall and membrane perturbing agents (congo red, caffeine and calcoflour white) at 30 °C (Figure 2b). A comparable result was obtained by incubating the spot assay plates at 37 °C, although at this temperature fungal growth was delayed and colonies were smaller (Figure S2). 

Finally, the ability to form pseudohyphae in vitro was investigated in the panel of mutant strains. As shown in Figure 2c, in presence of serum and at a temperature of 37 °C, pseudohyphae were observed in all *C. parapsilosis* mutant strains tested with no differences between wild type and mutant strains. Under the same conditions, *C. albicans* SC5314 control strain produced true hyphae.

### 3.3. CpALS4790 and CpALS0660 are Differently Involved in the Adhesion Process of C. parapsilosis to HBECs

**Figure 3:**
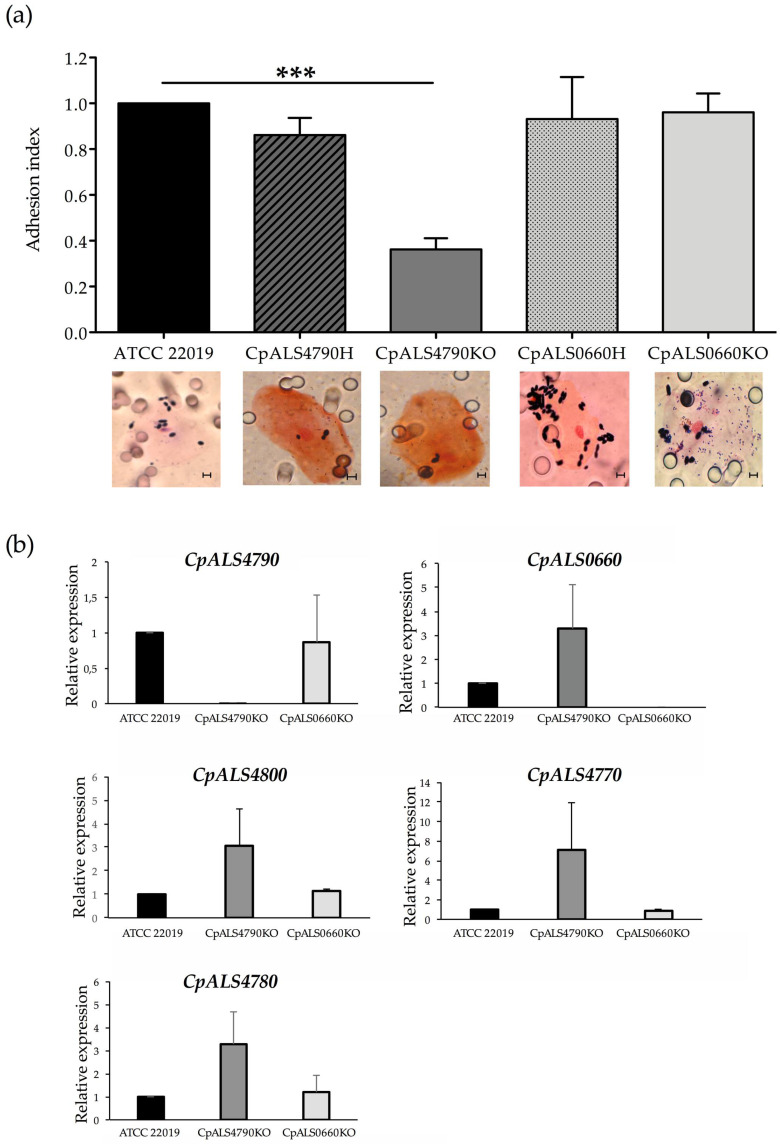
Adhesion to human epithelial buccal cells and *ALS* gene expression analysis. (**a**) *C. parapsilosis* wild type and mutant strains were tested for their ability to adhere to human buccal epithelial cells (HBECs). Bars represent adhesion index mean + standard error of mean. At least 3 biological replicates were used. ***p < 0.001. Representative micrographs showing Gram-stained *C. parapsilosis* blastoconidia of wild type and mutant strains adhered to a buccal cell are depicted below the graph (magnification = 1000×, scale bar denotes 10 μm). (**b**) Real-time RT PCR analysis performed on wild type and null mutant strains of *CpALS4790* and *CpALS0660* following co-incubation with HBECs. Transcriptional levels of the *CpALS* genes were evaluated for each strain and compared to wild type strain. Actin expression levels were used as an internal control for each strain.

An adhesion assay was performed in order to evaluate the role of *CpALS4790* and *CpALS0660* in the adhesion process of *C. parapsilosis* to biotic surfaces. As illustrated in Figure 3a, the deletion of one or both copies of *CpALS0660* did not impair *C. parapsilosis* adhesion ability to HBECs. Conversely, a significant decrease in the adherence to buccal cells was observed in the *CpALS4790* null mutant strain in comparison to the wild type strain, with more than 60% reduction in the mean adhesion index (*p* < 0.001). The impact of the deletion of *CpALS4790* and *CpALS0660* on the transcriptional profile of the five *CpALS* genes was investigated in the panel of mutant strains co-incubated with HBECs as previously described. Briefly, quantitative analysis of levels of expression of *C. parapsilosis ALS* genes was performed following a 45 min co-incubation with HBECs at 37 °C. As expected, no *CpALS4790* or *CpALS0660* transcripts were observed, confirming the deletion of the desired gene in each studied mutant strain. No significant changes in *ALS* gene family expression were observed in mutant strains (Figure 3b), further confirming that the reduced adhesion observed for the *CpALS4790* mutant strains was due to the loss of both *CpALS4790* gene copies and not to an overall change in the Als protein profiles.

### 3.4. CpALS4790 and CpALS0660 Contribute to Cell Adhesion and Pathogenesis in a Murine Model of Vaginal Candidiasis.

**Figure 4:**
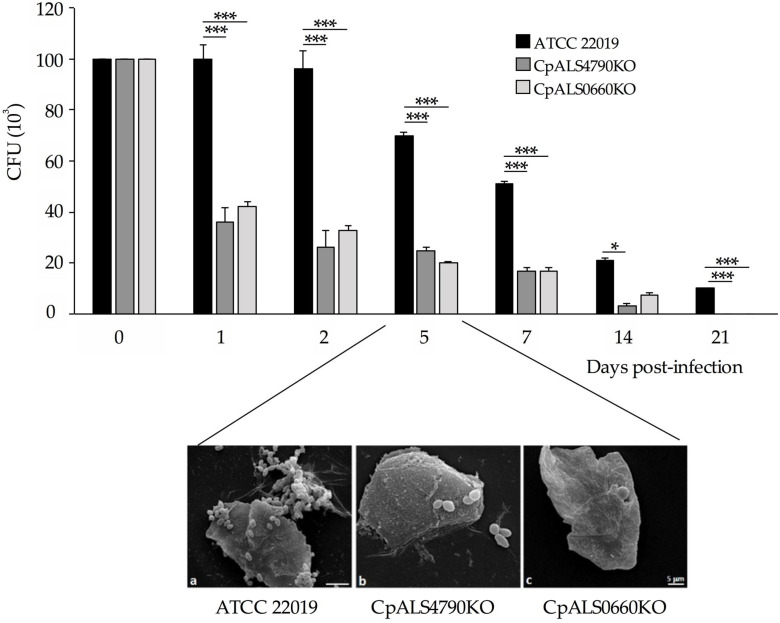
Analysis of the pathogenic potential of the *CpALS4790* and *CpALS0660* mutant strains in an in-vivo model of murine vaginal candidiasis. The pathogenicity of the mutant collection was tested in a murine model of vaginal candidiasis. Five BALB/c mice for each strain (WT, CpALS4790KO, CpALS0660KO) were intravaginally infected with 10^6^ yeast cells/20 μL. Bars represent mean values ± standard error of the mean of *C. parapsilosis* CFUs recovered from vaginal fluids at different time points. ***p < 0.0001, *p < 0.05. SEM micrographs of vaginal scrubs collected at five days post-infection show the presence of *C. parapsilosis* yeast cells of wild type and mutant strains adhered to vaginal epithelial cells. Magnification = 4550×, scale bar denotes 5 μm.

The pathogenic potential of the *CpALS4790* and *CpALS0660* mutants was assessed in an in vivo model of murine vaginal candidiasis. Following vaginal challenge, BALB/c mice were monitored for fungal vaginal burdens over a period of four weeks. As shown in Figure 4, the number of CFUs recovered from mice infected with null mutants was significantly lower with respect to those collected from wild type infected animals as early as 24 h post-infection. A significant reduction in the vaginal burden of mice infected with null mutant strains was maintained throughout the entire course of the infection. This finding clearly indicates that the deletion of *CpALS4790* and of *CpALS0660* genes results in a reduced ability to colonize and persist in the vaginal mucosa. In agreement with these results, scanning electron microscopy images (Figure 4) at five days post-infection clearly shows numerous *C. parapsilosis* yeast cells of wild type adhered to vaginal epithelial cells. By contrast, no or rare fungal cells of *CpALS4790* and *CpALS0660* null mutant strains were detected on murine vaginal cells by SEM observations. 

## 4. Discussion

The *Candida parapsilosis ALS*-like gene family has been recently described and the protein structure dissected for each member by in silico studies [8,12]. However, functional studies have been performed on three genes out of the five components of the family, by both deletion or CRISPR-Cas9 gene editing strategies [17,19,20]. 

In this study, the role of the two functionally still uncharacterized genes (*CpALS4790* and *CpALS0660*) in *C. parapsilosis* virulence and pathogenicity was assessed through the generation of mutant strains using the SAT1-flipper cassette system [15]. Although deletion of one *CpALS0660* allele was readily achieved, a strain lacking the second allele was not identified despite screening hundreds of transformants. To overcome this obstacle and to force the inactivation of the remaining *CpALS0660* allele, a new disruption cassette targeting a more internal upstream homology region was generated. Transformation with the new disruption cassette led to the generation of a null mutant strain, lacking both copies of *CpALS0660*. This type of difficulty in making homozygous strains have already been described by Zhao et al. [21], when *C. albicans ALS2* was selected as a target for mutagenesis. The authors circumvented this obstacle by placing the remaining *ALS2* allele under control of the inducible *C. albicans MAL2* promoter. Similar hurdles were also encounter by Green et al. [22] with *ALS2* gene inactivation, where only one clone in 800 showed integration of a GFP cassette in the correct locus. *CpALS0660* is the only gene of the *C. parapsilosis ALS* gene family located on another chromosome (Chr 4). We speculate that the different gene disruption efficiencies between the two alleles of *CpALS0660* may be the result of different chromatin accessibility, likely caused by silencing epigenetic factors. An example of this type of phenomena is provided by the *Saccharomyces* Genome Deletion Project (SGDP) [23], which was aimed at systematically replacing each yeast open reading frame (ORF) with the kanMX cassette. This approach allowed the construction of a gene knockout library covering 6000 genes that have been extensively used for gene function studies. Out of 528 genes that were not included in the mutant collection, 321 failed to be deleted using the kanMX disruption cassette [24]. An interesting study published by Chen and colleagues [24] demonstrated a statistically significant correlation between these ORFs and the presence of H3K36me3, an epigenetic modification of histone protein H3, which is commonly associated with gene silencing in yeast. In this regard, chromatin immunoprecipitation (ChIP) to detect the epigenetic landscape of *CpALS0660* would help us gain an insight into the reasons behind the failed attempt of gene deletion of the remaining wild type allele. Phenotypic analysis of wild type, heterozygous and null mutant strains obtained in the present study confirmed that the deletion of *CpALS4790* or *CpALS0660* did not affect the growth rate of the yeast in YPD medium. The same results were obtained when the growth ability was tested on solid media supplemented with cell-wall perturbing agents, with results comparable to those observed for the wild type strain. Moreover, the ability to switch from a yeast phase to a pseudohyphal form was not impaired. These findings are in agreement with what was previously observed for the *CpALS4770*, *CpALS4780* and *CpALS4800* null mutant strains, suggesting that the absence of these functional adhesins does not directly alter cell-wall structure or morphogenesis [13,14]. By contrast, as observed in one of our previous works, the contextual inactivation of both *CpALS4770* and *CpALS4780* led to the generation of elongated cells with a tendency to form cellular aggregates. This peculiar phenotype had a strong impact on the virulence traits of the double mutant strain, by increasing both the ability to form biofilms and to adhere on human buccal epithelial cells (HBECs) [13]. These findings suggest that compensatory mechanisms in the cell-wall composition may occur in response to the contextual lack of multiple adhesins or a specific combination of Als proteins. Generation of a different combination of *CpALS* mutant strains may help to shed light on this matter. For example, a decrease in the pseudohyphal formation was observed when the entire *ALS* gene family of *C. orthopsilosis* was inactivated through CRISPR-Cas9 gene editing [13].

The role of *CpALS4790* and *CpALS0660* in the adhesion process was assessed in an in vitro model of co-incubation of yeast cells in the presence of HBECs, as previously described [13,14,16,17,18]. Interestingly, the adhesion assay performed on *CpALS4790* mutant collections revealed a significant reduction in the adhesion ability of the null mutant strain to HBECs, with a >60% decrease of adhesion index if compared with the wild type strain. This data suggests a direct role for *CpAls4790*p in *C. parapsilosis* adhesion to buccal cells, as already described for other *C. parapsilosis* Als proteins such as *CpAls4770p* and *CpAls4800p* [13,14]. By contrast, no significant differences could be detected in the adhesive properties between the wild type and *CpALS0660* null mutant strain, suggesting that this gene may not be primarily involved in the adhesion process to HBECs, at least in the experimental conditions tested. A similar result was obtained when *CpALS4780* mutant was previously tested under the same conditions [13], suggesting that a wider range of experimental conditions and/or ligands may help to fully understand the contribution of these two *ALS* genes in the adhesion ability of *C. parapsilosis*. Interestingly, preliminary results on the adhesion properties of *C. orthopsilosis* null mutant strain lacking both copies of *CoALS800*, a syntenic gene of *CpALS0660,* showed no statistically significant difference in the adhesion ability if compared with the wild type parental strain (unpublished results). A similar finding was also described when gene disruption was performed on *C. albicans ALS2* and *ALS4*: wild type and null mutant strains adhered to buccal cells to the same extent, but the mutant’s deficit in adhesion was evident when the assay was performed on endothelial cells [21]. In order to evaluate potential compensatory mechanisms, we further investigated the effect of *CpALS4790* and *CpALS0660* gene deletion on the expression profiles of the other *CpALS* genes following co-incubation with HBECs. Real-time RT PCR experiments confirmed the lack of *CpALS4790* and *CpALS0660* transcripts in the respective mutant strains and did not show any significant changes in the expression level of the other *CpALS* gene family. This is in accordance with previous findings [13,14], where *CpALS4800*, *CpALS4770* and *CpALS4780* single deletions did not impair the expression profile of the remaining *ALS* genes. However, we cannot exclude the possibility that in mutant strains bearing multiple *ALS* deletions the expression of the other *ALS* genes may be affected. Finally, in order to highlight the role of *CpALS4790* and *CpALS4780* in a mucosal model of infection, wild type and null mutant strains were tested in a murine model of vaginal candidiasis. A statistically significant reduction in the number of the CFUs recovered from BALB/c mice intravaginally infected with both *CpALS4790* and *CpALS0660* null mutant strains was observed if compared with the number of CFUs recovered from mice infected with the wild type strain. These results clearly indicate that both CpALS4790p and CpALS0660p contribute to the pathogenesis of murine vaginal candidiasis. 

## 5. Conclusions

The overall results presented in this study complete the functional characterization of the *C. parapsilosis ALS* gene family by the generation of single mutant strains lacking the two remaining uncharacterized *ALS* genes, *CpALS4790* and *CpALS0660*. Phenotypic analysis of the null mutant strains indicated the involvement of *CpALS4790*, but not *CpALS0660*, in the adhesion ability of *C. parapsilosis* to human buccal epithelial cells. Notably, both genes contributed to the pathogenesis of *C. parapsilosis* when tested in a murine model of vaginal candidiasis. Future research will be aimed at further exploring the role of *C. parapsilosis ALS* genes in the adhesion process through testing additional potential ligands and using the CRISPR-Cas9 technology for the generation of multiple edited strains and their subsequent characterization in in vitro and in vivo models.

## Supplementary Materials

The following are available online at https://www.mdpi.com/2309-608X/6/2/86/s1, Figure S1: *CpALS4790* and *CpALS0660* PCR screening of recombinant clones. Figure S2: Growth ability of the wild type and *C. parapsilosis* mutant strains in solid media with or without cell-wall perturbing agents incubated at 37°C. Table S1: *C. parapsilosis* strains used and generated in this study. Table S2: Primers used in this study.

## References

[1] Hoyer L.L., Cota E. (2016). *Candida albicans* Agglutinin-Like Sequence (Als) Family Vignettes: A Review of Als Protein Structure and Function. Front. Microbiol..

[2] Hoyer L.L., Scherer S., Shatzman A.R., Livi G.P. (1995). *Candida albicans ALS1*: Domains related to a Saccharomyces cerevisiae sexual agglutinin separated by a repeating motif. Mol. Microbiol..

[3] Tavanti A., Davidson A.D., Gow N.A., Maiden M.C., Odds F.C. (2005). *Candida orthopsilosis and Candida metapsilosis* spp. nov. to replace *Candida parapsilosis* groups II and III. J. Clin. Microbiol..

[4] Bliss J.M. (2015). *Candida parapsilosis*: An emerging pathogen developing its own identity. Virulence.

[5] Guinea J. (2014). Global trends in the distribution of *Candida* species causing candidemia. Clin. Microbiol. Infect..

[6] Ward T.L., Dominguez-Bello M.G., Heisel T., Al-Ghalith G., Knights D., Gale C.A. (2018). Development of the Human Mycobiome over the First Month of Life and across Body Sites. MSystems.

[7] Ghannoum M.A., Jurevic R.J., Mukherjee P.K., Cui F., Sikaroodi M., Naqvi A., Gillevet P.M. (2010). Characterization of the oral fungal microbiome (mycobiome) in healthy individuals. PLoS Pathog..

[8] Butler G., Rasmussen M.D., Lin M.F., Santos M.A., Sakthikumar S., Munro C.A., Rheinbay E., Grabherr M., Forche A., Reedy J.L. (2009). Evolution of pathogenicity and sexual reproduction in eight *Candida* genomes. Nature.

[9] Riccombeni A., Vidanes G., Proux-Wera E., Wolfe K.H., Butler G. (2012). Sequence and analysis of the genome of the pathogenic yeast Candida orthopsilosis. PLoS ONE.

[10] Pryszcz L.P., Nemeth T., Saus E., Ksiezopolska E., Hegedusova E., Nosek J., Wolfe K.H., Gacser A., Gabaldon T. (2015). The Genomic Aftermath of Hybridization in the Opportunistic Pathogen Candida metapsilosis. PLoS Genet..

[11] Lombardi L., Zoppo M., Rizzato C., Bottai D., Hernandez A.G., Hoyer L.L., Tavanti A. (2019). Characterization of the *Candida orthopsilosis* agglutinin-like sequence (*ALS*) genes. PLoS ONE.

[12] Oh S.H., Smith B., Miller A.N., Staker B., Fields C., Hernandez A., Hoyer L.L. (2019). Agglutinin-Like Sequence (*ALS*) Genes in the *Candida parapsilosis* Species Complex: Blurring the Boundaries Between Gene Families That Encode Cell-Wall Proteins. Front. Microbiol..

[13] Zoppo M., Di Luca M., Franco M., Rizzato C., Lupetti A., Stringaro A., De Bernardis F., Schaudinn C., Barrasa M.I., Bottai D. (2020). CpALS4770 and CpALS4780 contribution to the virulence of *Candida parapsilosis*. Microbiol. Res..

[14] Bertini A., Zoppo M., Lombardi L., Rizzato C., De Carolis E., Vella A., Torelli R., Sanguinetti M., Tavanti A. (2016). Targeted gene disruption in *Candida parapsilosis* demonstrates a role for *CPAR2_404800* in adhesion to a biotic surface and in a murine model of ascending urinary tract infection. Virulence.

[15] Reuss O., Vik A., Kolter R., Morschhauser J. (2004). The SAT1 flipper, an optimized tool for gene disruption in *Candida albicans*. Gene.

[16] Zoppo M., Lombardi L., Rizzato C., Lupetti A., Bottai D., Papp C., Gacser A., Tavanti A. (2018). CORT0C04210 is required for *Candida orthopsilosis* adhesion to human buccal cells. Fungal Genet. Biol..

[17] Zoppo M., Luca M.D., Villarreal S.N., Poma N., Barrasa M.I., Bottai D., Vyas V.K., Tavanti A. (2019). A CRISPR/Cas9-based strategy to simultaneously inactivate the entire *ALS* gene family in Candida orthopsilosis. Future Microbiol..

[18] Bertini A., De Bernardis F., Hensgens L.A., Sandini S., Senesi S., Tavanti A. (2013). Comparison of *Candida parapsilosis*, *Candida orthopsilosis*, and *Candida metapsilosis* adhesive properties and pathogenicity. Int J. Med. Microbiol..

[19] Lombardi L., Oliveira-Pacheco J., Butler G. (2019). Plasmid-Based CRISPR-Cas9 Gene Editing in Multiple *Candida* Species. MSphere.

[20] Lombardi L., Turner S.A., Zhao F., Butler G. (2017). Gene editing in clinical isolates of *Candida parapsilosis* using CRISPR/Cas9. Sci. Rep..

[21] Zhao X., Oh S.H., Yeater K.M., Hoyer L.L. (2005). Analysis of the *Candida albicans* Als2p and Als4p adhesins suggests the potential for compensatory function within the Als family. Microbiology.

[22] Green C.B., Zhao X., Yeater K.M., Hoyer L.L. (2005). Construction and real-time RT-PCR validation of *Candida albicans* PALS-GFP reporter strains and their use in flow cytometry analysis of *ALS* gene expression in budding and filamenting cells. Microbiology.

[23] Winzeler E.A., Shoemaker D.D., Astromoff A., Liang H., Anderson K., Andre B., Bangham R., Benito R., Boeke J.D., Bussey H. (1999). Functional characterization of the *S. cerevisiae* genome by gene deletion and parallel analysis. Science.

[24] Chen M., Licon K., Otsuka R., Pillus L., Ideker T. (2013). Decoupling epigenetic and genetic effects through systematic analysis of gene position. Cell Rep..

