# Supplementary material for: Role of CpALS4790 and CpALS0660 in Candida parapsilosis Virulence: Evidence from a Murine Model of Vaginal Candidiasis
Source: J Fungi (Basel). 2020 Jun 12;6(2):86. doi: 10.3390/jof6020086 (PMC7345767; doi:10.3390/jof6020086)
Supplement: Supplementary file 1 [file jof-06-00086-s001.pdf]

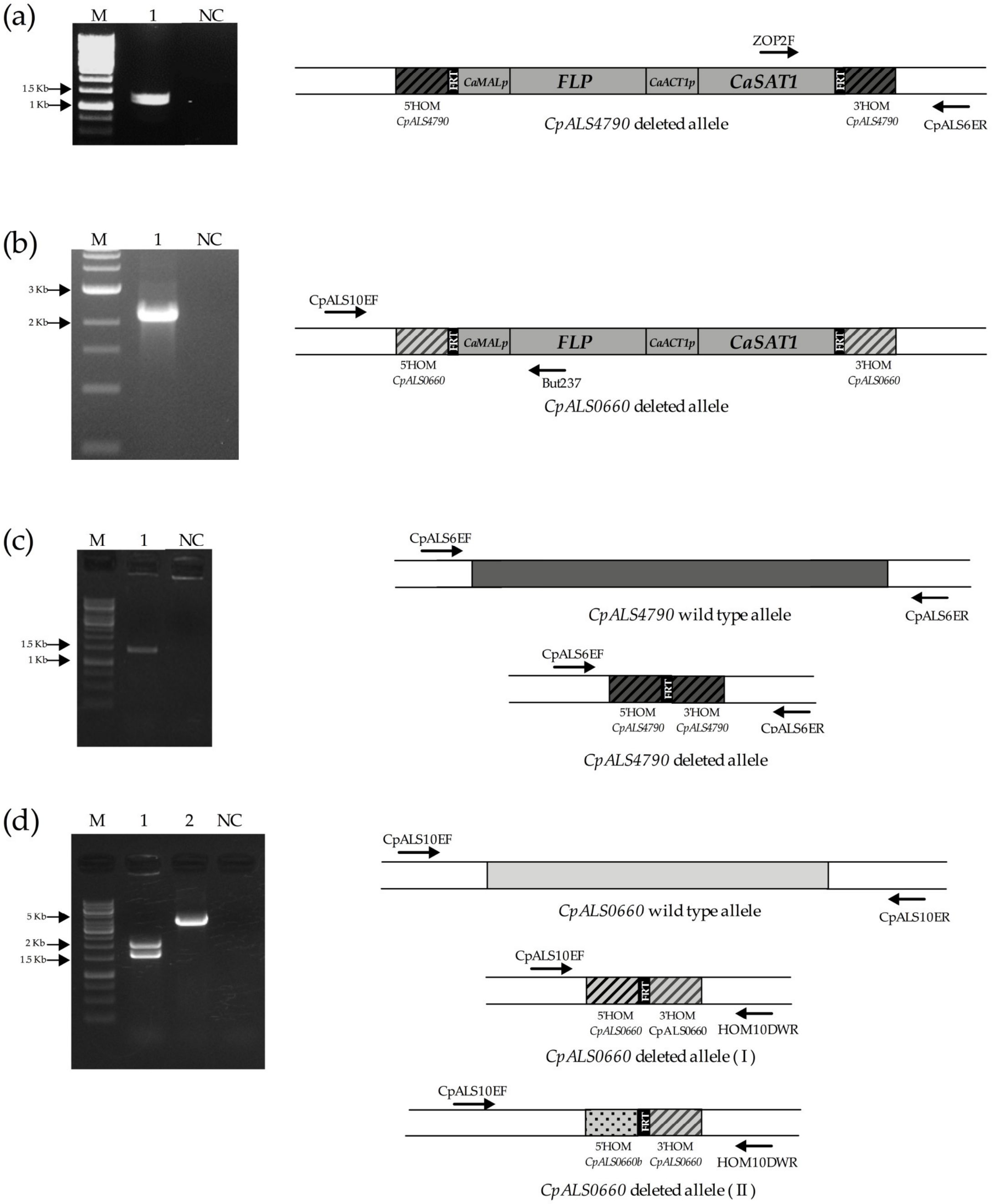

Supplementary Figure 1: *CpALS4790* and *CpALS0660* PCR screening of recombinant clones. (**a**) Correct integration of the SAT1-flipper cassette in *CpALS4790* locus was performed using primers ZOP2F/CpALS6ER, yielding a PCR product of 1,3 Kb. Figure shows a representative gel obtained when CpALS4790KOC strain was used as template. M: 1 Kb Gene-ruler marker (Thermo Scientific); 1: CpALS4790KOC; NC: Negative control. (**b**) Correct integration of the SAT1-flipper cassette in *CpALS0660* locus was performed using primers CpALS10EF/But237, yielding a PCR product of 2,2 Kb. Figure shows a representative gel obtained when CpALS0660KOC strain was used as template. M: 1 Kb Gene-ruler marker (Thermo Scientific); 1: CpALS0660KOC; NC: Negative control. (**c**) Amplification of the *CpALS4790* locus with primers CpALS4790EF/CpALS4790ER of 1,4 Kb in CpALS4790KO. M: 1 Kb Gene-ruler marker (Thermo Scientific); 1: ATCC 22019; 2: CpALS4790KO; NC: negative control. (**d**) Amplification of the *CpALS0660* locus with primers CpALS0660EF/HOM10DWR generated a fragment of 4,6 Kb in ATCC 22019 and two fragments of 1,6 Kb and

2,1 Kb in CpALS0660KO mutant strain. M: 1 Kb Gene-ruler marker (Thermo Scientific); 1: CpALS0660KO; 2: ATCC 22019; NC: negative control.

Table S1: *C. parapsilosis* strains used and generated in this study.

Strain name	Parent	Genotype
ATCC 22019 (WT)	/	*C. parapsilosis* parental strain
CpALS4790HC	ATCC 22019	CpALS4790/cpALS4790Δ::SAT1-FLP
CpALS4790H	CpALS4790HC	CpALS4790/cpALS4790Δ::FRT
CpALS4790KOC	CpALS4790H	cpALS4790Δ::SAT1-FLP/cpALS4790Δ::FRT
CpALS4790KO	CpALS4790KOC	cpALS4790Δ::FRT/ cpALS4790Δ::FRT
CpALS0660HC	ATCC 22019	CpALS0660/cpALS0660Δ::SAT1-FLP
CpALS0660H	CpALS0660HC	CpALS0660/cpALS0660Δ::FRT
CpALS0660KOC	CpALS0660H	cpALS0660Δ::SAT1-FLP/cpALS0660Δ::FRT
CpALS0660KO	CpALS0660KOC	cpALS0660Δ::FRT/cpALS0660Δ::FRT

Table S2: Primers used in this study.

Primer name	Sequence (5′ → 3′)	Usage
**Primer used for the amplification of homology regions**		
HOM6UPF*	TATC**GGGCCC**CAAAACAGTACACGAGAAAG	Upstream homology region of *CpALS4790*
HOM6UPR*	CATA**CTCGAG**ATGTTCCTGTTGCTTCCATA	Upstream homology region of *CpALS4790*
HOM6DWF*	TACT**CCGCGG**GCGGCGTCAAACACAGAATC	Downstream homology region of *CpALS4790*
HOM6DWR*	TATA**GAGCTC**GCCCCTGTCTGTGATTGAACG	Downstream homology region of *CpALS4790*
HOM10UPF*	ATAA**GGGCCC**GATTTCCAAAGCGTCATTGC	Upstream homology region of *CpALS0660*
HOM10UPR*	GATC**CTCGAG**ATGAAGGTGCCAGGTTTTTG	Upstream homology region of *CpALS0660*
HOM10UPF1*	ATAA**GGGCCC**AGAGACATTGCTTGACGGCA	Upstream homology region of *CpALS0660* (internal cassette)
HOM10UPR1*	GATC**CTCGAG**CCAGCAGCATTTGTGCTTG	Upstream homology region of *CpALS0660* (internal cassette)
HOM10DWF*	TATT**CCGCGG**CCCAAGCATACAACAACAAC	Downstream homology region of *CpALS0660*
HOM10DWR*	GTCC**GAGCTC**AAAATGGTACAAGTGGAGGA	Downstream homology region of *CpALS0660*
**Primer used for screening of the mutant collection**		
CpALS6EF	CATAACACACATTTCCATAG	*CpALS4790* external primer for the upstream correct integration of the disruption cassette
CpALS6ER	CTTGAGGGCTTCGTCTACGC	*CpALS4790* external primer for the downstream correct integration of the disruption cassette

CpALS10EF	GAGATTGCGTTTACATCGTGCT	*CpALS0660* external primer for the upstream correct integration of the disruption cassette
CpALS10ER	AAAATGGTACAAGTGGAGGA	*CpALS0660* external primer for the downstream correct integration of the disruption cassette
ZOP2F	TCTGATGAAGACTCTGCTTGC	SAT1-flipper cassette internal primer
But237 [1]	GCTGTTCCGTTATGTGTAATCATCC	SAT1-flipper cassette internal primer
**Primer used for RT-qPCR analysis**		
ACT1F	AGTGTGACTTGGATGTCAGAAAGGAATTGT	Actin primer for qRT-PCR
ACT1R	ACAGAGTATTTTCTTTCTGGTGGAGCA	Actin primer for qRT-PCR
CPAG_05314 F	GGGATCAGCAAATTCTGTCGA	*CpALS0660* primer for qRT-PCR
CPAG_05314 R	CCAGCGGTAAAACATTGGGA	*CpALS0660* primer for qRT-PCR
CPAG_00368 F	TGTCCTCGACAACTCCAGCTT	*CpALS4770* primer for qRT-PCR
CPAG_00368 R	GGTTCTAAAATGGGTGGAATGG	*CpALS4770* primer for qRT-PCR
CPAG_05056 F	AAAGTCACCACCACCGAGGTT	*CpALS4800* primer for qRT-PCR
CPAG_05056 R	CGGCGCAGATGTGCTAATG	*CpALS4800* primer for qRT-PCR
CPAG_05054 F	TCGAGTTCCTAATGGTGCAG	*CpALS4790* primer for qRT-PCR
CPAG_05054 R	CCTTCTTCACCCCAGTTTTG	*CpALS4790* primer for qRT-PCR
CPAG_00369F	AACGTCCAACAGGTCAAGTG	*CpALS4780* primer for qRT-PCR
CPAG_00369R	CTCCCCATTTTATTGATTGTGAG	*CpALS4780* primer for qRT-PCR

*Restriction sites are highlighted in bold.

[1] Ding, C.; Butler, G. Development of a gene knockout system in Candida parapsilosis reveals a conserved role for BCR1 in biofilm formation. *Eukaryot Cell* **2007**, *6*, 1310-1319, doi:10.1128/EC.00136-07.
